# Evaluation of different grafting materials for alveolar cleft repair in the context of orthodontic tooth movement in rats

**DOI:** 10.1038/s41598-021-93033-x

**Published:** 2021-06-30

**Authors:** Stephan Christian Möhlhenrich, Kristian Kniha, Zuzanna Magnuska, Benita Hermanns-Sachweh, Felix Gremse, Frank Hölzle, Gholamreza Danesh, Ali Modabber

**Affiliations:** 1 Department of Orthodontics, University of Witten/Herdecke, Alfred-Herrhausen Str. 45, 58455 Witten, Germany; 2 Department of Oral and Maxillofacial Surgery, University Hospital of Aachen, Pauwelsstraße 30, 52074 Aachen, Germany; 3 Institute for Experimental Molecular Imaging, RWTH Aachen University, Forckenbeckstraße 55, 52074 Aachen, Germany; 4 Implant Pathology, ZBMT, Campus Melaten, Pauwelsstraße 17, 52074 Aachen, Germany

**Keywords:** Oral anatomy, Experimental models of disease, Preclinical research

## Abstract

To minimize the postoperative risks posed by grafting autologous transplants for cleft repair, efforts are being made to improve grafting materials for use as potential alternatives. The aim of this study was to compare the bone graft quality of different bone substitutes including the gold standard autografts during the healing processes after cleft repair in the context of orthodontic treatment. In 21 Wistar rats, a complete, continuity-interrupting cleft was created. After 4 weeks, cleft repair was performed using autografts from the hips’ ischial tuberosity, human xenografts, or synthetic bone substitutes [beta-tricalcium phosphate (β-TCP)/hydroxyapatite (HA)]. After another 4 weeks, the first molar movement was initiated in the reconstructed jaw for 8 weeks. The bone remodeling was analyzed in vivo using micro-computed tomography (bone mineral density and bone volume fraction) and histology (new bone formation). All the grafting materials were statistically different in bone morphology, which changed during the treatment period. The β-TCP/HA substitute demonstrated less resorption compared to the autologous and xenogeneic/human bone, and the autografts led to a stronger reaction in the surrounding bone. Histologically, the highest level of new bone formation was found in the human xenografts, and the lowest was found in the β-TCP/HA substitute. The differences between the two bone groups and the synthetic materials were statistically significant. Autografts were confirmed to be the gold standard in cleft repair with regard to graft integration. However, parts of the human xenograft seemed comparable to the autografts. Thus, this substitute could perhaps be used as an alternative after additional tissue-engineered modification.

## Introduction

Different types of bone grafts have been introduced for alveolar cleft repair, such as autografts (e.g., iliac crest, cranium, tibia, rib, and mandibular symphysis), allografts or xenografts, and synthetic bone substitutes (e.g., bioceramics, polymers, or biocomposites)^1–3^. Due to their osteogenic, osteoinductive, and osteoconductive properties, grafts from the iliac crest are considered the gold standard for cleft repair^4^. However, these autografts pose some unique risks and may cause postoperative morbidities, such as pain, hematoma, and delayed ambulation, which may lead to limited bone supply, the demand for an additional donor site, and the associated inherent susceptibility to resorption in the long term, among others^5–10^. Therefore, grafting materials with different origins were continuously improved to enhance the clinical outcome and reduce the postoperative morbidity^3,4,11^.

Different rat models have been presented for cleft research^12–20^, but most models are not in accord with the clinical situation in which the defect is covered by the epithelial lining. In these cleft models the bone defect is created and filled in the same operation. However, this is not in accordance to the clinical bony situation^21^. For this, a bone defect must first be created, and after defect healing is achieved with the mucosal lining, the grafting material must be placed in a second-stage surgery.

With regard to the cleft location in the rat models, a distinction is made between the mid-palate cleft in the anterior part of the maxilla^12–14^ and the alveolar cleft in the central^13,15,16^ or posterior^17–20^ maxilla. The defects in the posterior part usually accompany extraction; that is, they are more like large extraction defects than complete interruptions of the alveolar ridge continuity as per the meaning of a cleft. Furthermore, only the posterior alveolar cleft allows a subsequent molar movement through the reconstructed jaw.

In this context, a new alveolar cleft model in rats was recently introduced, which offers a completely maxillary interruption that is covered by the epithelial lining and allows a subsequent orthodontic tooth movement after cleft repair^22^.

The effects of the different bone substitutes and their long-term outcomes are unreliable, especially in the context of subsequent orthodontic tooth movement. Sun et al. reported that orthodontic movement into an alveolar cleft bone graft area could strengthen the bone reconstruction process owing to the mechanical pressure from the orthodontic stimuli, which enhances the bone remodeling of the graft bone reconstructs into the autogenous bone. Furthermore, it provides a bone matrix for shifting teeth^18^. However, the researchers did not compare autografts with other kinds of grafting materials. In this context, Ru et al. compared a synthetic bone substitute based on a mixture of hydroxyapatite (HA) and beta-tricalcium phosphate (β-TCP) with a bovine xenograft in a corresponding alveolar defect model in rats^20^. They found the least amount of tooth movement, volumes and craters of root resorption, and the highest bone volume fraction (BV/TV), trabecular number, and mean trabecular thickness in the synthetic bone group^19^. Human allografts are also acceptable alternatives for cleft repair as reduced operation time, shortened hospital stays, and less graft resorption over time were reported when they were used^23^. However, there is limited information about the healing process after cleft repair using human allografts.

Recently, Kamal et al. reported in a meta-analysis that tissue-engineered bone substitutes are as effective as autogenous bone in reducing the volume of the cleft. They concluded that this creates a viable option of eliminating the need for a second surgical site with its associated postoperative morbidity^6^. However, almost all of these examinations do not take into account the often-necessary subsequent orthodontic tooth movement, which may negatively affect the healing process or be disturbed by the presence of a bone substitute.

Therefore, the aim of the present basic research was to compare the healing process of three different grafting materials (autografts, human xenografts, and synthetic biphasic calcium phosphate bone substitutes) after cleft repair and in the context of orthodontic treatment.

## Materials and methods

### Experimental design and laboratory animals

**Figure 1:**
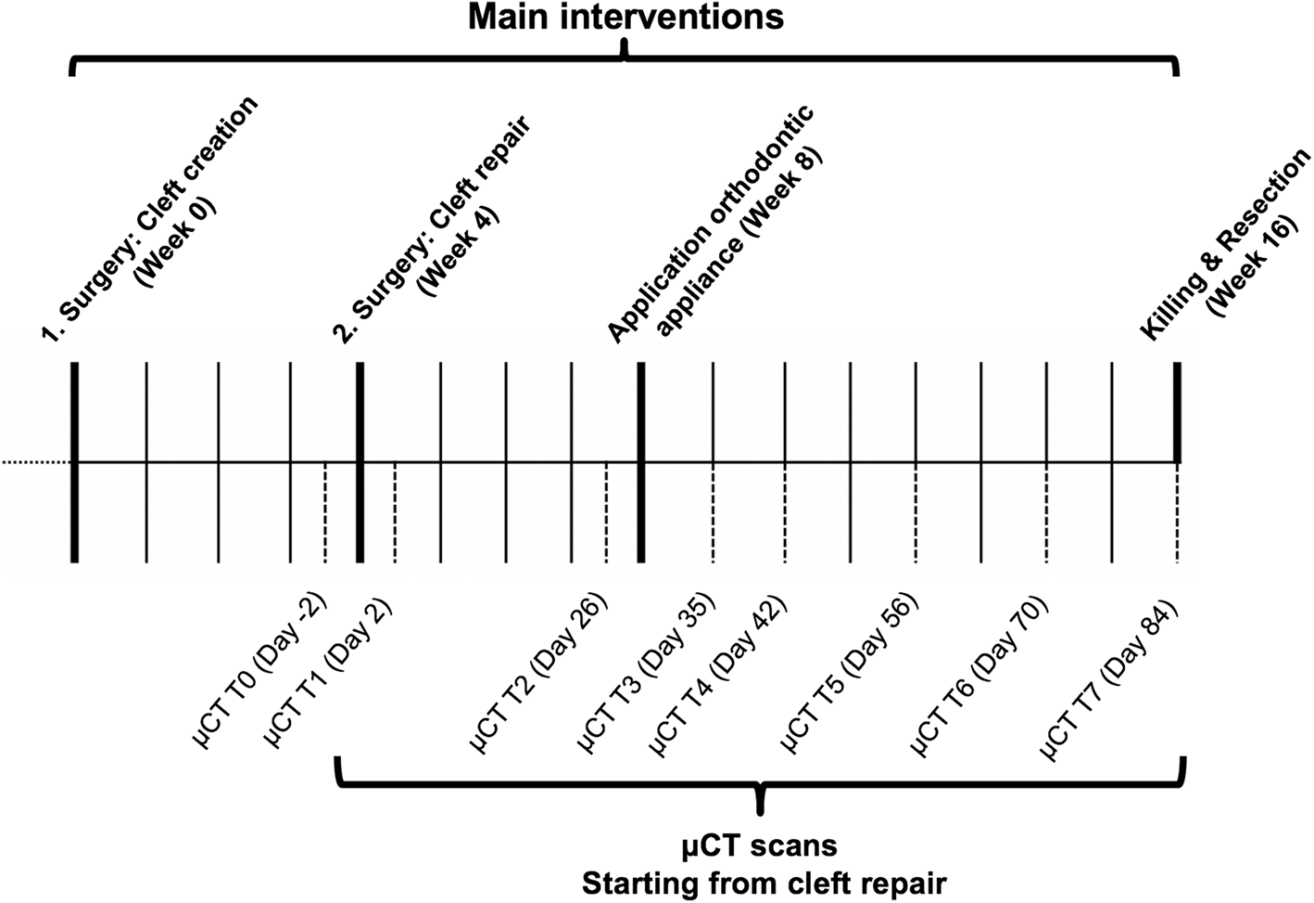
Timeline of the animal cleft research procedure: the thick, solid lines represent the experimental measures under intraperitoneal injection, such as cleft creation, cleft repair, application of orthodontic appliances, killing, and resection, while the dotted lines represent the radiological follow-up monitoring in µ-CT under isoflurane anesthesia.

A new alveolar cleft model in rats was developed^22^. Therefore, a priori sample size calculation with regard to root resorption during orthodontic tooth movement in different cleft repairs was performed using one-way ANOVA (analysis of variance). The sample size calculation was based on the mean apical root resorption reported by Ru et al.^19^ in animals were xenogeneic and synthetic bone substitutes were used for cleft repair. The sample size estimation relying on the large observed effect (0.0605 mm^3^ vs. 0.089 mm^3^) and the corresponding difference between the xenogeneic and autologous bones was assumed to be half of the difference between the xenogeneic and synthetic bone substitutes. The common standard deviation was considered to be 0.01, which corresponds to 10% of the highest value for mean root resorption reported by Ru et al.^19^. The level of significance was set at 0.0125 to reflect the four different regions investigated by Ru et al.^19^, and a 1.3538 effect size was characterized to reach at least 80% power in a one-way ANOVA model with three groups. The study design envisaged the use of seven animals per cleft-repair-type group, including two rats for dropout over a treatment period of 16 weeks (Fig. 1).

All the experiments were conducted in accordance with the German Animal Welfare Act (Tierschutzgesetz, TSchG) and the EU Directive (2010/63/EU). The study protocol was approved by the Governmental Animal Care and Use Committee (Reference No. 81-02.04.2018.A342; Landesamt für Natur, Umwelt und Verbraucherschutz Recklinghausen, Nordrhein-Westfalen, Germany; dated 11.01.2019). The study protocol also complied with the ARRIVE Guidelines^24^ and the Guide for the Care and Use of Laboratory Animals. All the animals were group-housed in filter-top cages (Type 2000, Tecniplast, Buguggiate, Italy), three per cage. Low-dust wood granulate was used as bedding (Rettenmeier Holding AG, Wilburgstetten, Germany) and as the cage enrichment nesting material (Nestlet, 14010, Plexx B.V., Elst, Netherlands).

All the animals (N = 21) were randomly determined from a random number table to three groups of seven animals each (N = 7) based on the kind of grafting material that was used for cleft repair: autologous bone from the hip, xenogeneic bone (human bone substitute material; maxgraft, botiss biomaterials, Zossen, Germany), or synthetic bone substitute material (β-TCP and HA; maxresorb, botiss biomaterials, Zossen, Germany). No including or excluding criteria were determined.

Experimental alveolar clefts were created at the left side of the upper jaws of 8-week-old male Wistar-HAN rats (Janvier Labs, Le Genest-Saint-Isle, France) with an average weight of 465 ± 34 g. A week before the animals were fed with a high-energy nutritional supplement (DietGel Boost, Clear H2O, Portland, USA) for the special food habituation. Four weeks later, a second surgery for cleft repair was performed in the same rats, which by then were already 12 weeks old and had an average weight of 504 ± 36 g. Another four weeks later, after bone consolidation, an orthodontic appliance was applied in all the rats, which by then were already 16 weeks old and had an average weight of 542 ± 32 g. After further 8 weeks of orthodontic treatment, the animals were killed at the age of 24 weeks. At this time, the average weight of all animals was 555 ± 42 g.

All these interventions were performed via general anesthesia with intraperitoneal injection of a combination of ketamine (80–100 mg/kg, Ketavet, Pfizer, Berlin, Germany) and medetomidine hydrochloride (0.15–0.25 mg/kg, Domitor, Orion Pharma, Espoo, Finland) combined with endotracheal intubation using a 15-gauge intravenous catheter for oxygen substitution. Buprenorphine (0.03–0.05 mg/kg, Temgesic, Indivior Limited, Berkshire, UK) was applied subcutaneously as an analgesic. Cefuroxime (15 mg/kg s.c., Fresenius, Bad Homburg, Germany) for antibiotic treatment was started at 24 h intervals for 7 days after the surgical interventions. Immediately after all the interventions, atipamezole hydrochloride (0.75 mg/kg, Antisedan, Orion Pharma, Espoo, Finland) was given as a reversing agent, and further analgesia was carried out if necessary with buprenorphine (0.03–0.05 mg/kg) for a maximum period of 5 days.

All the animals were put back in their cages after the surgical and orthodontic treatments under intensive monitoring, and were observed until their full recovery. After these interventions, the rats were given special soft food (DietGel Boost, Clear H2O, Portland, USA) for 7 days as refinement, followed by a standard diet (rat/mouse maintenance #V1534-300, 10 mm; ssniff Spezialdiäten GmbH, Soest, Germany) and water ad libitum. Finally, after the last imaging, the animals were killed through cervical dislocation under general anesthesia, and samples were taken for further histological processing.

### Surgical interventions

All surgical procedures were performed as previous described in anesthesia using a combination of ketamine (80–100 mg/kg, Ketavet, Pfizer, Berlin, Germany) and medetomidine hydrochlorid (0.15–0.25 mg/kg, Domitor, Orion Pharma, Espoo, Finland)^22^. Buprenorphine (0.03–0.05 mg/kg, Temgesic, Indivior Limited, Berkshire, UK) for analgesia and cefuroxime (15 mg/kg s.c., Fresenius, Bad Homburg, Germany) for antibiotic treatment were administered subcutaneously. Additionally, to ensure sufficient oxygenation, endotracheal intubation was performed by the use of a 16-gauge intravenous catheter.

**Figure 2:**
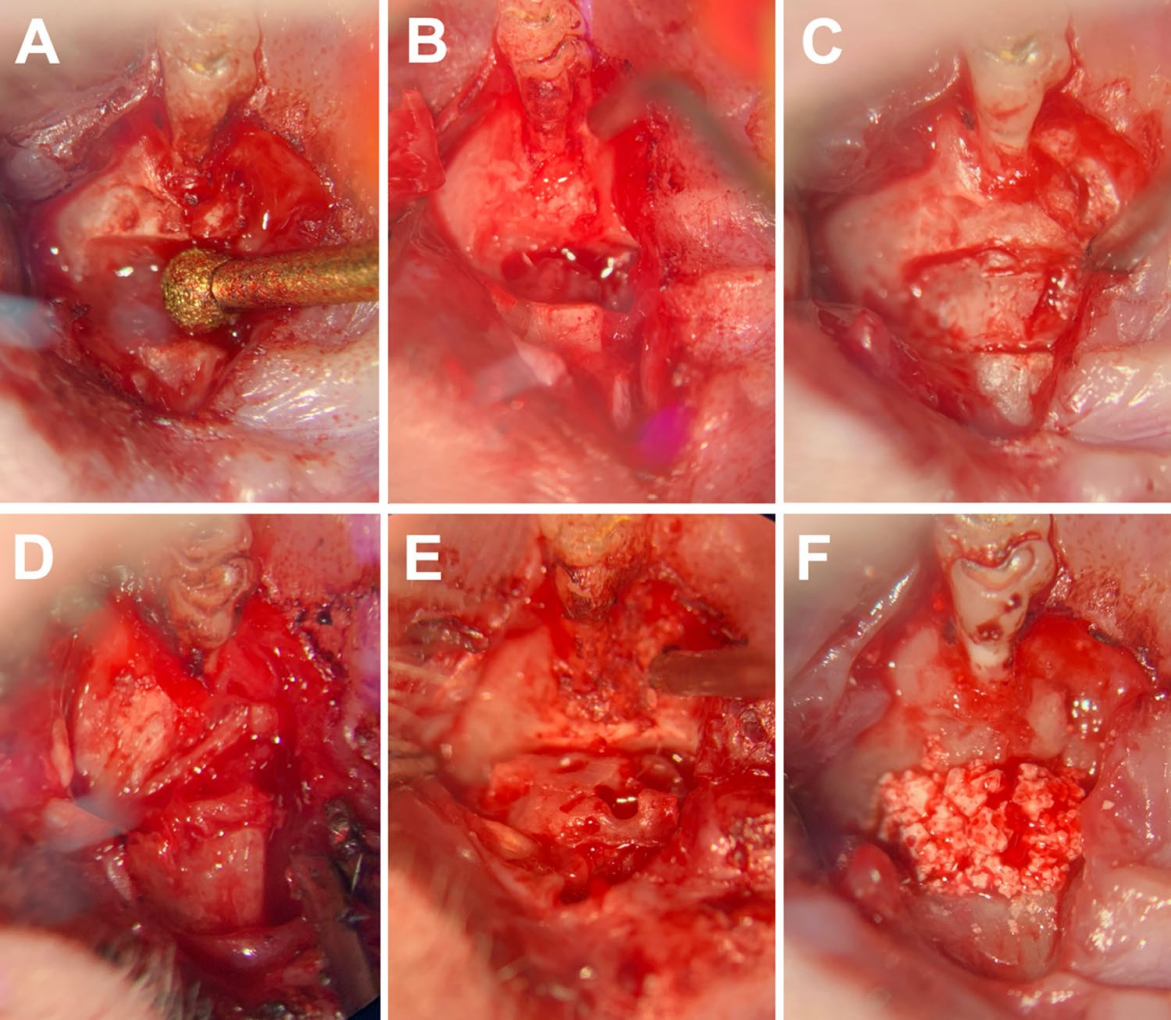
View of the operative situs of the left maxilla in supine position (magnification × 10): first molar above, mouth tip below: (**A**) artificial alveolar cleft creation using an ultrasonic device; (**B**) alveolar cleft with intact mucosa to the maxillary sinus and nasal passage; and (**C**) artificial alveolar cleft filled with bone wax. Re-entry and cleft repair were performed with (**D**) autograft from the ischial tuberosity of the hip, (**E**) human xenograft, or (**F**) β-TCP/HA bone substitute material.

All the animals were placed in a supine position, and their mouths were disinfected (Cutasept, BODE Chemie GmbH, Hamburg, Germany). After an incision in the attached gingiva down to the bone between the first molar and the anterior part of maxilla, the soft tissue was deflected. Then an osteotomy with a diameter of 1.7 mm was carried out between the roots of the incisor and the first molar from the vestibule and the palatine foramen using an ultrasonic device (insert OT5, Mectron s.p.a., Carasco, Italy) under irrigation with a sterile physiologic solution (Fig. 2A,B). Afterwards, bone wax (Bonewax, Ethicon—Johnson & Johnson Medical GmbH, Norderstedt, Germany) was applied to preserve the artificial cleft (Fig. 2C). Finally, wound closer was done using continuous resorbable sutures (7/0 Vicryl, Ethicon, Johnson & Johnson Medical, Somerville, NJ, USA).

In the second operation the same anesthetic protocol was used. The soft tissue was deflected using the previously used approach. In the animals with jaw reconstruction using autologous bone, this was done before grafting from the left ischial tuberosity in the hip^25^. After the exposure and removal of the bone wax, the bone surrounding the cleft was refreshed. Thereafter, the maxilla was reconstructed using autologous bone from the hip, xenogeneic bone (human bone substitute material), or synthetic bone substitute (β-TCP and HA), respectively (Fig. 2D–F). Finally, the wound was closed again through continuous resorbable sutures (7/0 Vicryl, Ethicon, Johnson & Johnson Medical, Somerville, NJ, USA).

### Orthodontic intervention

**Figure 3:**
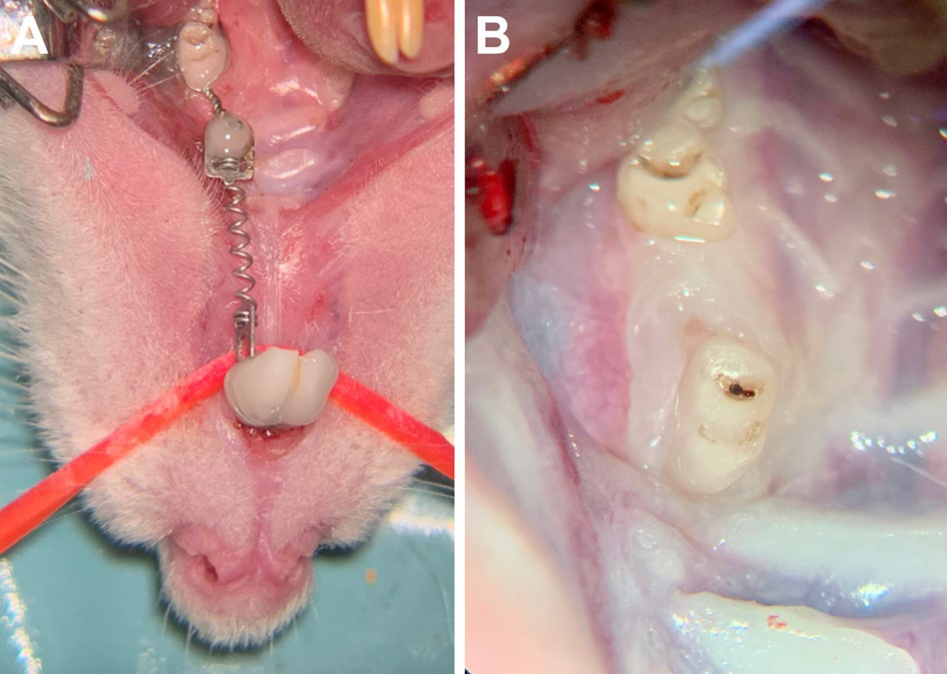
(**A**) Applied orthodontic appliance based on a 0.14 N nickel–titanium closed coil tension spring fixed between the first molar and the incisors using tension springs after conditioning of the teeth through acid etching using 39% phosphonic acid and bonding agent and dental composite (magnification × 4). (**B**) Anterior moved first molar after 8 weeks of orthodontic tooth movement (magnification × 6).

For orthodontic tooth movement, a 0.14 N nickel–titanium closed coil tension spring (33-54495, PSM Medical Solutions GmbH, Gunningen, Germany) was installed between the incisors and the upper-left first molar (according to Kirschneck et al.)^26–28^ by wire ligature (Ø 0.01″) and dental composite (Venus flow, Kulzer GmbH, Hanau, Germany), using an acid-etching technique (Fig. 3A). Here, an about 0.14 N continuous force was applied. Additionally, to prevent damage to the spring, the lower incisors were ground during the radiological examinations. The orthodontic tooth movement was finished in 8 weeks (Fig. 3B).

### Microfocus computed tomography (µCT) analysis

**Figure 4:**
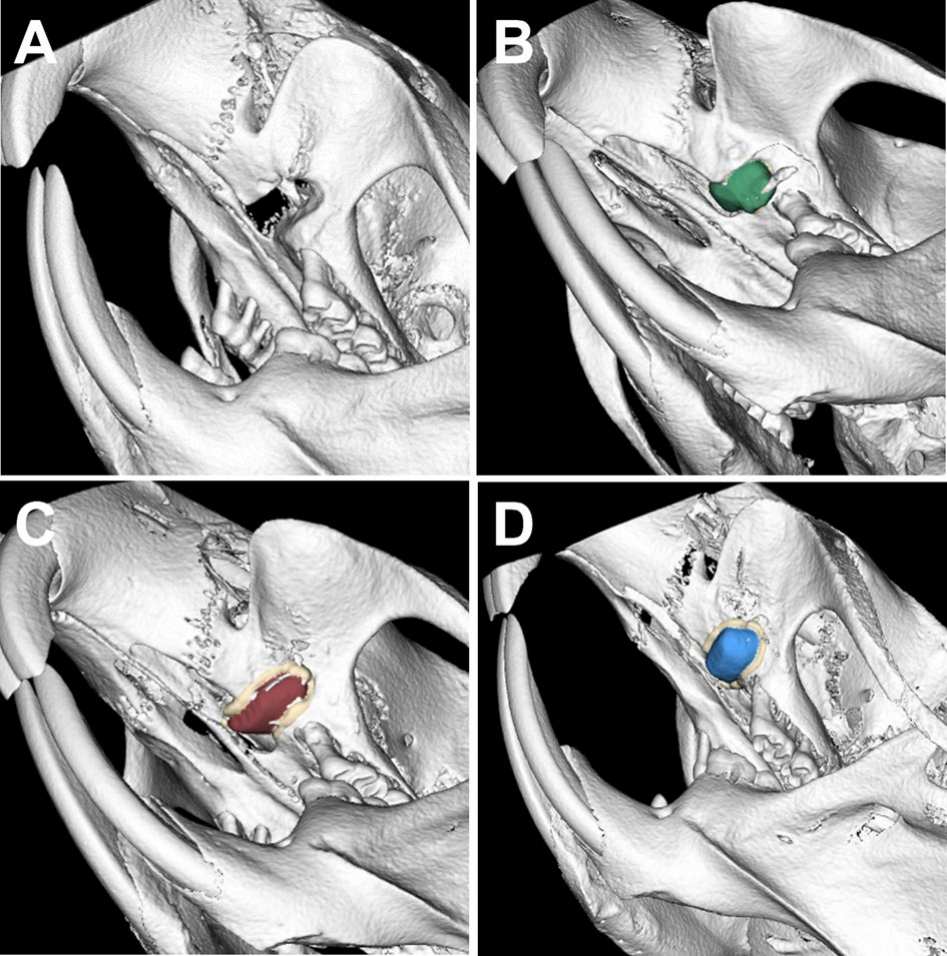
Three-dimensional micro-CT volume rendering after (**A**) cleft creation (µCT T0) and immediately after cleft repair (µCT T1) using (**B**) autologous bone (green), (**C**) xenogeneic/human bone (red), and (**D**) synthetic (β-TCP/HA) bone substitute (blue) and the surrounding alveolar bone (beige area).

Two days before (T0) and after (T1) cleft repair and 2 days before (T2) and 7 days (T3) after orthodontic-appliance installation, the rats were imaged with an in vivo µCT system (U-CT OI, MILabs, Utrecht, Netherlands) under general anesthesia using isoflurane [induction: 5 vol% isoflurane + 5 L O_2_/min; maintenance: 2 vol% isoflurane + 2 L O_2_/min] (Abbott GmbH & Co. KG, Wiesbaden, Germany). Additional imaging was performed every 2 weeks (T4–T7) for radiographic follow-up analysis of the reconstructed maxilla. The radiological analysis was based on ultra-focus magnification through 360° rotation at 0.75° increments with 0.3 s/degree, and the data were reconstructed at a 40 µm isotropic voxel size. For analysis, the data were downsampled by binning them to a 80 µm voxel size, thus improving the visual appearance of the scans. The images were evaluated using cross-sectional slices and the rendered three-dimensional iso-surfaces (Fig. 4).

**Figure 5:**
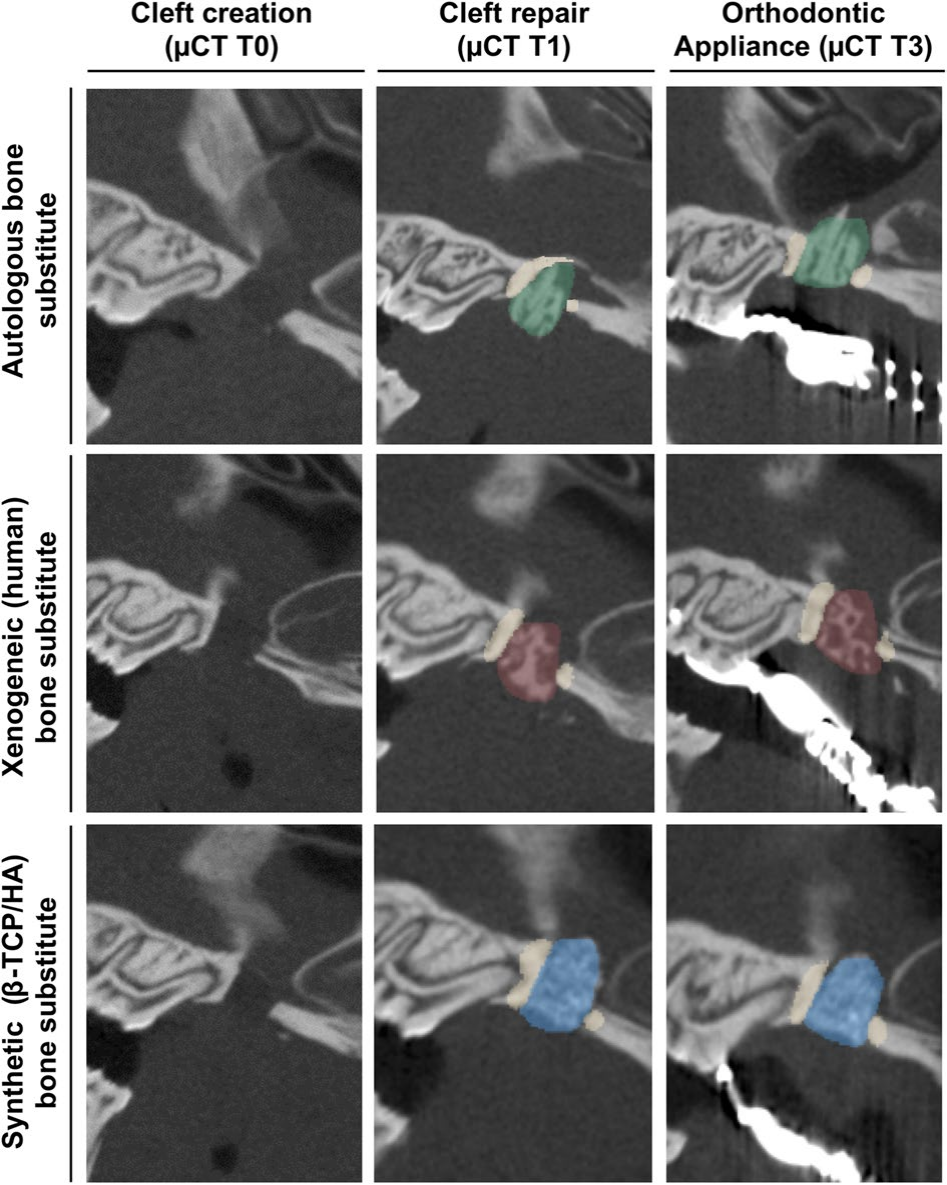
Sagittal view of the CT scans after cleft creation (µCT T0) and cleft repair with and without an orthodontic appliance (µCT T1, T3) for analyzing the bone quality of the augmented bone in the cleft (green area: autologous bone; red area: xenogeneic bone; blue area: synthetic bone substitute) and the surrounding alveolar bone (beige area) (magnification × 40).

For the analysis of the reconstructed maxilla, the grafted materials were segmented in micro-CT images using all the anatomical planes. Group affiliation was not known during the analysis. Afterwards, a coat with a fixed 10 voxel thickness was computed around the segment using morphological operation^29^. Then the bone tissue was segmented within the coat volume via thresholding. The reconstructed maxilla and the surrounding bone were then analyzed in terms of bone mineral density (BMD) and bone volume fraction (BV/TV) (Fig. 5).

### Histomorphometric analysis

**Figure 6:**
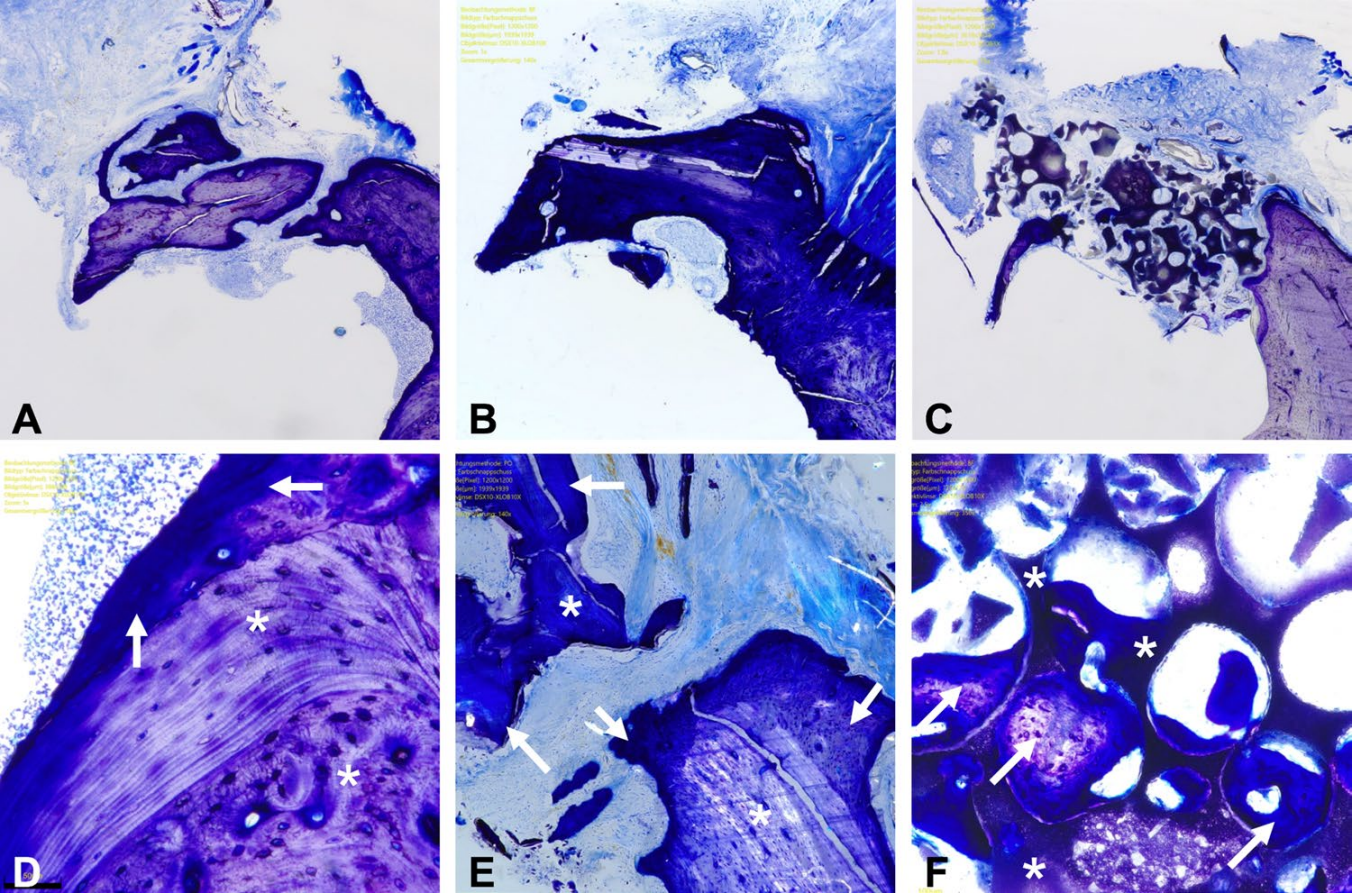
Histological cross-section (toluidine blue stains) through the reconstructed jaw 84 days after cleft repair using autologous bone (**A**,**D**), xenogeneic/human bone (**B**,**E**), and synthetic tricalcium phosphate/hydroxyapatite bone substitute (**C**,**F**): overview: (**A**–**C**) × 100 magnification; detailed view: (**D**–**F**) up to × 350 magnification, persistent bone/substitute (*), new bone formation (arrows).

The samples were stored in 4% formalin (neutrally buffered with methanol) for 48 h (Otto Fischar GmbH & Co. KG, Saarbrücken, Germany), and decalcification was carried out for approximately 4 weeks at 37 °C by storing the samples in 20-fold-volume ethylenediaminetetraacetic acid (EDTA, MolDecalcifer, Menarini, Florence, Italy), which was changed every 2 days. After being rinsed with tap water, the samples were stored for 24 h in 5% sucrose with phosphate-buffered saline (100 ml; 5 g sucrose). Then the samples were shock frozen in liquid nitrogen and embedded (TissueTek, Sakura, Alphen, Netherlands). Subsequently, 5- to 7-μm-thick cross-sections from the area immediately in front of the first molar were cut, mounted on Superfrost slides, and dried. The samples were fixed in acetone for 10 min and then stained with toluidine blue according to the routine protocols (Fig. 6A–C).

A specialized pathologist analyzed the tissue structures via light microscopy with software support (OLYMPUS Stream software, OLYMPUS digital microscope DSX-1000, Olympus Hamburg, Germany). Group affiliation was not provided to the investigator. To more precisely differentiate the proportions of different bone quality (lamellar bone vs. woven bone) and newly formed bone from the bone substitute materials, the samples were additionally examined using polarization optics. The augmented area and the area of the newly formed bone inside and outside the substitute were determined to quantify the osseous build-up or bone substitute material that was still present (Fig. 6D–F).

### Statistical analysis

The BMD and BV/TV in the grafting material as well as surrounding alveolar cleft bone were reported for the primary outcomes in the radiological analysis and the amount of persistent graft material or new bone formation in histologic analysis. All data were tested for normal distribution using the Shapiro–Wilk test. A two-way analysis of variance (ANOVA) was performed for bone substitute (autograft, human xenograft, synthetic bone substitute), and time of the radiological examination (µCT 1–7) or the amount of histological hard tissue (grafting material, overall/inside/outside new bone formation), respectively. The model also included post hoc comparisons between the groups with the use of Tukey multiple comparison test. The level of significance was set at p ≤ 0.05 by using the statistical program Prism (version 8; GraphPad Software Inc). All results are expressed as mean ± standard deviation values.

### Ethics approval and consent to participate

The experimental animal study protocol was approved by the Governmental Animal Care and Use Committee (Reference No.: 81-02.04.2018.A342; Landesamt für Natur, Umwelt und Verbraucherschutz Recklinghausen, Nordrhein-Westfalen, Germany; dated: 11.01.2019). The study protocol conforms to the ARRIVE Guidelines and with the Guide for the Care and Use of Laboratory Animals. All applicable international, national, and/or institutional guidelines for the care and use of animals were followed.

## Results

### Surgical procedure and experimental observations

After the optimization of the surgical procedure, the cleft repair with all the three bone substitutes was good and effective in handling. While the autologous and xenogeneic (human) bones were sufficiently fixed through the press-fit technique, the synthetic bone substitute material (β-TCP/HA) was carefully installed under condensation. Two animals died in association with second surgery while six animals remained in the autologous- and xenogeneic-bone groups while seven remained in the synthetic-bone-substitute group.

In the radiological follow-up imaging during the tooth movement, a total of 11 broken devices were found, with two found in one animal. The loss rate was distributed almost equally among the three groups (autograft: 3 of 6, human xenograft: 3 of 6, and synthetic bone substitute 4 of 7). Reattachment was carried out under intraperitoneal general anesthesia after continuous transition from isoflurane anesthesia.

### Micro-CT imaging

**Figure 7:**
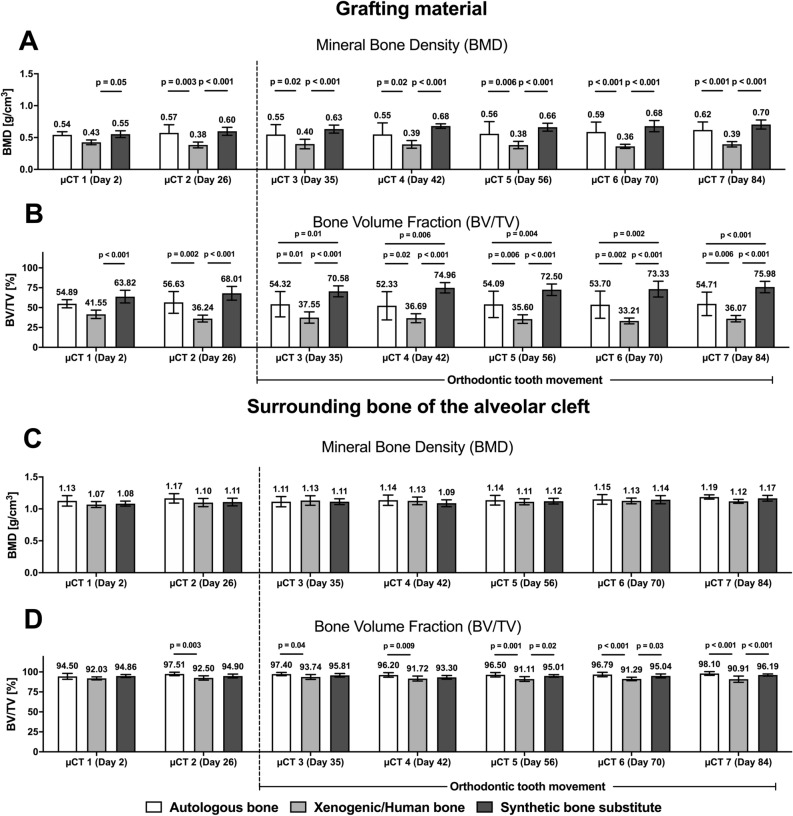
Radiological changes of the bone mineral density (BMD) and bone volume fraction (BV/TV) in the grafting materials (**A**,**B**) and in the bone surrounding the cleft (**C**,**D**) of the maxillary reconstruction: column bars of the mean values and p-values for the comparisons between the three different materials at seven points in the 84-day healing period after cleft repair.

In the three groups, bone bridging and defect filling were found in all the μCT images. Mean values and standard deviation (SD) of bone mineral density (BMD) and bone volume fraction (BV/TV) in the grafting materials and the corresponding surrounding clef bone are shown in the Table 1. The p-values of the significant differences of the corresponding statistical comparisons are demonstrated in the line chart diagram in Fig. 7.

**Table 1:** Mean, minimum and maximum values with standard deviation (SD) of bone mineral density (BMD) and bone volume fraction (BV/TV) in three different grafting materials and the corresponding surrounding clef bone in rats over an investigation period of 84 days.

Substitute	Imaging (µCT)	Graft								Surrounding cleft bone							
		BMD (g/cm^3^)				BV/TV (%)				BMD (g/cm^3^)				BV/TV (%)			
		Mean	SD	Min	Max	Mean	SD	Min	Max	Mean	SD	Min	Max	Mean	SD	Min	Max
Autograft	1 (Day 2)	0.54	0.05	0.49	0.60	54.89	5.07	49.56	61.68	1.13	0.08	1.01	1.21	94.50	3.70	87.56	97.41
	2 (Day 26)	0.57	0.13	0.41	0.71	56.63	13.66	38.37	73.13	1.17	0.07	1.07	1.25	97.51	2.15	93.55	99.61
	3 (Day 35)	0.55	0.16	0.26	0.66	54.32	15.89	25.66	69.72	1.11	0.08	1.03	1.23	97.40	2.02	94.41	99.77
	4 (Day 42)	0.55	0.18	0.25	0.70	52.33	17.78	23.22	68.68	1.14	0.08	1.04	1.23	96.20	2.84	92.82	99.14
	5 (Day 56)	0.56	0.19	0.25	0.72	54.09	16.57	30.13	70.48	1.14	0.08	1.06	1.24	96.50	2.82	93.26	99.39
	6 (Day 70)	0.59	0.16	0.38	0.75	53.70	17.12	32.14	73.33	1.15	0.08	1.04	1.22	96.79	2.77	93.13	98.90
	7 (Day 84)	0.62	0.13	0.44	0.74	54.71	14.74	36.56	72.13	1.19	0.03	1.14	1.23	98.10	2.36	93.33	99.50
Human xenograft	1 (Day 2)	0.43	0.04	0.38	0.49	41.55	5.27	33.34	48.88	1.07	0.05	0.99	1.13	92.03	1.90	89.76	94.51
	2 (Day 26)	0.38	0.05	0.33	0.46	36.24	4.25	31.76	43.38	1.10	0.07	1.03	1.20	92.50	2.67	88.53	96.59
	3 (Day 35)	0.40	0.08	0.31	0.52	37.55	7.09	30.23	49.82	1.13	0.08	1.03	1.22	93.74	3.03	90.63	97.67
	4 (Day 42)	0.39	0.06	0.33	0.49	36.69	5.67	30.95	45.25	1.13	0.06	1.06	1.22	91.72	3.15	88.29	97.28
	5 (Day 56)	0.38	0.06	0.31	0.47	35.60	5.46	29.72	43.29	1.11	0.05	1.06	1.19	91.11	3.00	87.90	96.38
	6 (Day 70)	0.36	0.03	0.33	0.40	33.21	3.66	28.49	37.52	1.13	0.05	1.06	1.19	91.29	2.18	88.31	94.60
	7 (Day 84)	0.40	0.04	0.33	0.45	36.07	3.99	29.81	41.52	1.12	0.03	1.07	1.16	90.91	3.91	87.14	97.58
Synthetic bone substitute	1 (Day 2)	0.55	0.05	0.45	0.61	63.82	7.98	50.82	74.38	1.08	0.04	1.04	1.15	94.86	1.86	91.91	97.70
	2 (Day 26)	0.60	0.06	0.48	0.66	68.01	8.66	55.02	77.03	1.11	0.06	1.05	1.22	94.90	2.39	92.35	99.05
	3 (Day 35)	0.63	0.06	0.50	0.68	70.58	6.86	57.43	77.82	1.11	0.05	1.07	1.20	95.81	2.23	91.58	98.87
	4 (Day 42)	0.68	0.04	0.65	0.72	74.96	6.55	71.18	82.52	1.09	0.05	1.05	1.15	93.30	2.34	91.61	95.98
	5 (Day 56)	0.66	0.06	0.53	0.72	72.50	7.21	59.86	80.68	1.12	0.05	1.06	1.18	95.01	1.64	92.13	96.89
	6 (Day 70)	0.68	0.09	0.49	0.73	73.33	9.95	52.86	83.34	1.14	0.06	1.07	1.26	95.04	2.49	91.54	98.95
	7 (Day 84)	0.71	0.07	0.55	0.76	75.98	7.16	63.20	84.21	1.17	0.05	1.11	1.25	96.19	1.25	94.75	97.96

With regard to the BMD, except at day 2 (µCT 1), statistically significant differences were found between the autologous and xenogeneic/human bones (p ≤ 0.02) and between the synthetic bone substitute and xenogeneic/human bone (p ≤ 0.05) for each measurement, but not between the autologous bone and the synthetic bone substitute (Fig. 7A). In contrast, the BV/TV values of all the groups showed statistically significant differences from day 35 (µCT 3) (p ≤ 0.01) (Fig. 7B).

Regarding the morphology of the bone surrounding the cleft, statistically significant differences in BV/TV were found between the groups, but no such differences were found in BMD (Fig. 7C,D). The difference between the autologous and xenogeneic/human bones (p < 0.04) occurred over the entire study period, and that between the xenogeneic bone and the synthetic bone substitute (p < 0.03) occurred from day 56 (µCT 5).

**Figure 8:**
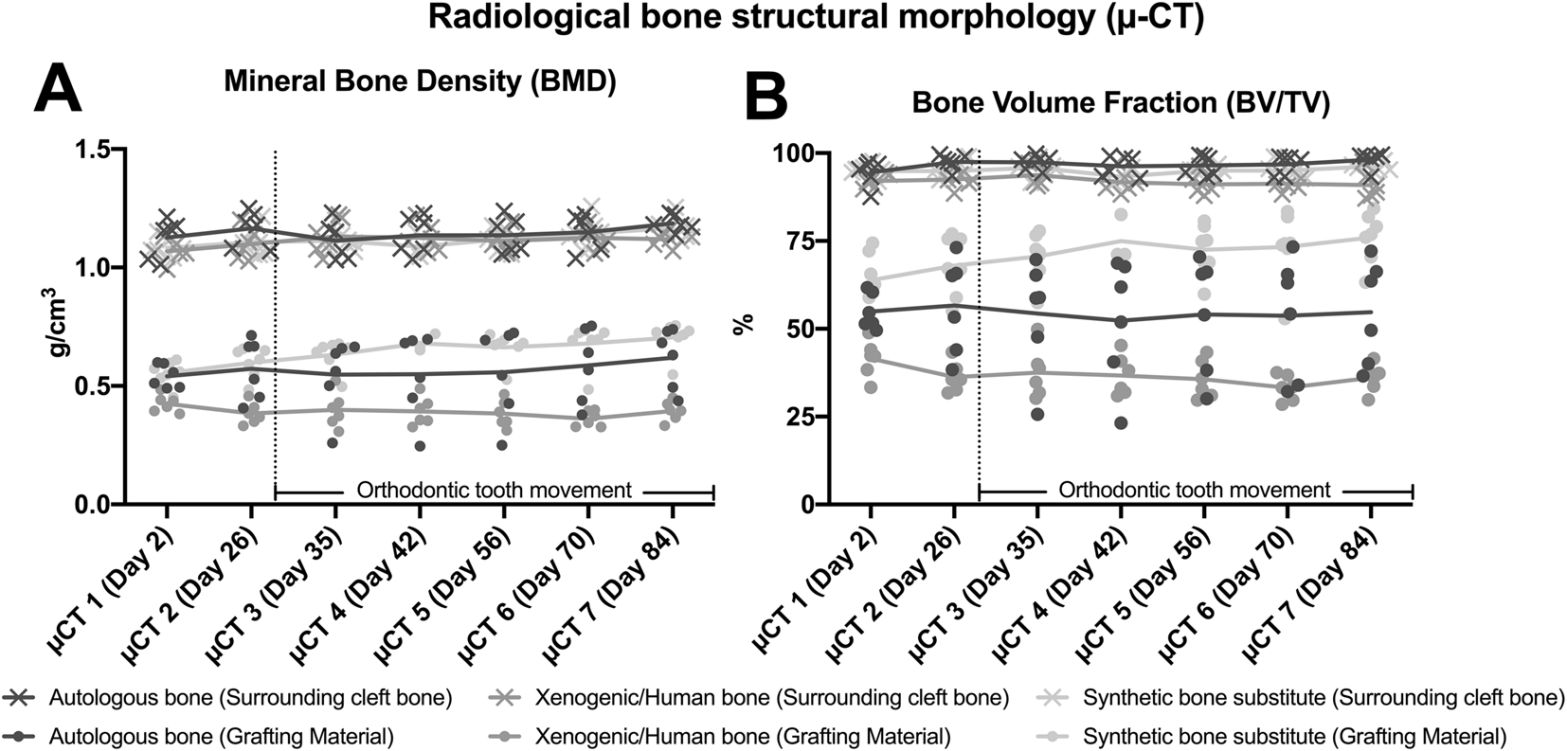
Radiological bone changes in the (**A**) bone mineral density (BMD) and (**B**) bone volume fraction (BV/TV) of the grafting materials and the bone surrounding the cleft in the maxillary reconstruction: line diagram of the mean values in the context of the bone structural morphology behavior over time.

During the investigation, no statistically significant changes in BMD were found between the initial and final bone morphologies (µCT 1 vs. µCT 7) in each group (autologous bone 0.54 ± 0.05 g/cm^3^ vs. 0.62 ± 0.13 g/cm^3^, p = 0.79; xenogeneic/human bone 0.43 ± 0.04 g/cm^3^ vs. 0.39 ± 0.04 g/cm^3^, p = 0.99; synthetic bone substitute 0.65 ± 0.07 g/cm^3^ vs. 0.67 ± 0.05 g/cm^3^, p = 0.99) (Fig. 8A). Even after the orthodontic tooth movement was initiated (µCT 2 vs. µCT 3), none of the grafting materials showed statistically significant changes in BMD (p > 0.95). The obtained BV/TV values correspond to these findings (µCT 1 vs. µCT 7: autologous bone 54.89 ± 5.07% vs. 54.71 ± 14.74%, p > 0.99; xenogeneic/human bone 41.55 ± 5.27% vs. 36.07 ± 3.99%, p = 0.97; synthetic bone substitute 63.82 ± 7.98% vs. 75.98 ± 7.16%, p = 0.29) (Fig. 8B).

No statistically significant differences were found between the initial and final bone morphologies (µCT 1 vs. µCT 7) within the bone surrounding the cleft for the BMD (autologous bone 1.13 ± 0.08 g/cm^3^ vs. 1.19 ± 0.04 g/cm^3^, p = 0.59; xenogeneic/human bone 1.07 ± 0.05 g/cm^3^ vs. 1.12 ± 0.03 g/cm^3^, p = 0.77; synthetic bone substitute 1.09 ± 0.04 g/cm^3^ vs. 1.17 ± 0.05 g/cm^3^, p = 0.13) and for the BV/TV (autologous bone 95.50 ± 3.70% vs. 98.10 ± 2.36%, p = 0.20; xenogeneic/human bone 92.03 ± 1.90% vs. 90.91 ± 3.91%, p = 0.99; synthetic bone substitute 94.86 ± 1.86% vs. 96.19 ± 1.25%, p = 0.96) (Fig. 8).

Statistically significant differences were found between all the grafting materials and the local surrounding bones (Table 2).

**Table 2:** p-values of comparisons between grafted material and corresponding the surrounding cleft bone depending on the healing process.

Grafted material	Grafted material vs. Surrounding cleft bone						
	µCT1	µCT2	µCT3	µCT4	µCT5	µCT6	µCT7
**BMD**							
Autograft	< 0.001	< 0.001	< 0.001	< 0.001	< 0.001	< 0.001	< 0.001
Human xenograft	< 0.001	< 0.001	< 0.001	< 0.001	< 0.001	< 0.001	< 0.001
β-TCP/HA substitute	< 0.001	< 0.001	< 0.001	< 0.001	< 0.001	< 0.001	< 0.001
**BV/TV**							
Autograft	< 0.001	< 0.001	< 0.001	< 0.001	< 0.001	< 0.001	< 0.001
Human xenograft	< 0.001	< 0.001	< 0.001	< 0.001	< 0.001	< 0.001	< 0.001
β-TCP/HA substitute	< 0.001	< 0.001	< 0.001	0.03	< 0.001	< 0.001	< 0.001

### Histomorphology analysis

**Figure 9:**
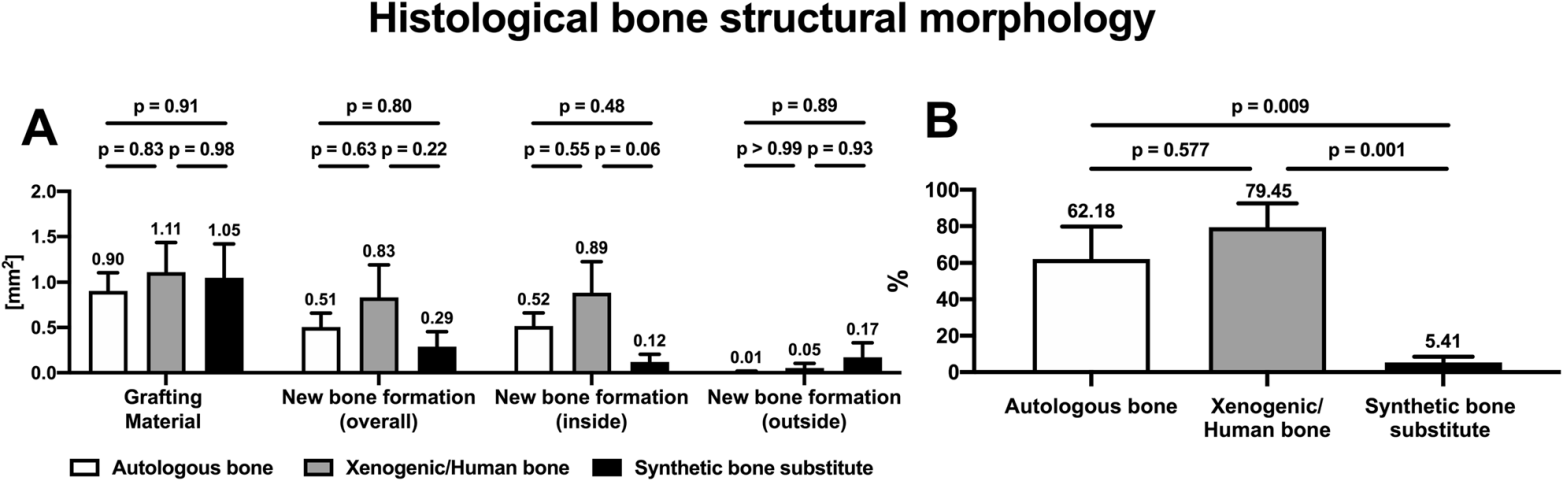
Results of the histological structural analysis of the reconstructed maxilla with regard to persistent grafting material and new bone formation: column bars of the mean values (**A**) or the corresponding percentage (**B**) and p-values for the comparisons of the three materials after the 84-day cleft repair healing period.

In all the histological samples, the proportions of the grafting substitutes were comparable to each other as there were no significant differences between the groups (p > 0.83) (Fig. 9). In general, the area of the recognizable applied grafting material in the autograft group was 0.90 ± 0.45 mm^2^, that in the xenogeneic/human bone group was 1.11 ± 0.79 mm^2^, and that in the synthetic substitute group was 1.05 ± 0.98 mm^2^.

The strongest new bone formation was found in the human xenograft, followed by the autograft and synthetic bone substitute (xenogeneic/human bone: 0.83 ± 0.88 mm^2^; autologous bone: 0.51 ± 0.34 mm^2^; synthetic substitute: 0.29 ± 0.43 mm^2^), but there were no statistically significant differences between the groups in this regard (p > 0.22).

In all the groups, more new bone formation took place within the applied grafting material than outside it. The highest new bone fusion occurred in the xenogeneic/human bone (79.45 ± 32.13%), and the lowest in the synthetic bone substitute (5.41 ± 8.06%). The bone integration in the autologous bone was about 62.18 ± 39.44%. The differences between the synthetic bone substitute and the autograft (p = 0.009) and between the synthetic bone substitute and the human xenograft (p = 0.001) were significant. However, there was no significant difference between the autologous and xenogeneic/human bones (p = 0.57).

## Discussion

Alveolar cleft repair aims at bone reconstruction that corresponds to the natural anatomy in terms of bone volume and quality. These cleft defects are unique with regard to the associated soft-tissue connection of the oral and nasal mucosa. In this context, various experimental cleft models in rats have been used to investigate cleft repair techniques and grafting materials^12–20^. However, these models did not coincide with a clinical cleft, where the epithelium lining is defective. Furthermore, it is not clear if they will allow an optional and adequate subsequent orthodontic tooth movement. This is because the constructed defect is too far away from the molar or because the alveolar cleft is not completely continuity interrupting. The present study design was thus developed; its cleft morphology is similar to those of human patients and it allows cleft repair using autologous bone grafts from a new donor site and a subsequent orthodontic tooth movement in the reconstructed area^22,25^. However, it must be critically noted that at the beginning some of the orthodontic appliances were lost in all three groups that means force-free time intervals up to 2 weeks. This represents a potential bias. However, the rate of loss was almost equally distributed among all three groups and at approximately the same time. Thus, it can be assumed that a possible impact from the loss of appliance has led to a similar influence in all three groups.

It must be noted that the present study design is limited in its translatory potential for a clinical setting. Although critical size defects, like alveolar clefts, in rat models have been very useful to understand the biology of cleft repair using grafting materials, the comparative age of the animals differs from that of humans. In literature, postnatal maturity for rats is termed as peri-adolescent phase beginning at day 49, while young adulthood period starting at day 70. In this study the artificial cleft osteotomy was performed around day 56 (8-week-old animals). Therefore, the rodents in the present study were in the pubertal age according to Sengupta^30^. However, the cleft repair was done around postnatal day 84 (12-week-old animals). At the moment the rodents were previously in the adolescent phase and even in adult period during orthodontic treatment. Furthermore, anatomical differences remain between a congenital cleft of the human maxilla and an iatrogenic produced jaw defect in the rat, even though the present model represents an improvement on previous study designs. A further limitation of the study is the usability of findings for allografts from animal studies, due the human processed bone substitute must be regarded as xenogeneic grafts in animals. Consequently, the collagen structures that still contained in the substitute can lead to immunological reactions^31^.

Autologous bone remains the gold standard for cleft repair and can be grafted from various donor sites^5–7^. However, bony autografts have some disadvantages, including limited bone supply, the demand for an additional donor site, the associated postoperative morbidity (pain, hematoma, and delayed ambulation), and an inherent susceptibility to resorption in the long term^5–9^. Therefore, various tissue-engineered bone substitutes have been proven to be good alternatives for promoting bone fusion and eliminating donor site morbidity^7,32–34^.

Calcium phosphate ceramics are commonly used in clinical practice as synthetic bone substitutes, and are contained in different alloplastic forms, such as calcium sulfate, tricalcium phosphate (TCP), or biphasic TCP (BTCP)^34,35^. For achieving sufficient β-TCP resorption properties, the ideal balanced ratio of HA and β-TCP ranges between 65:35 and 55:45^36,37^. In the context of animal cleft research, De Ruiter et al. compared the healing process of β-TCP and autogenous bone grafts in a goat model and reported that the bone healing of β-TCP was similar to that of the autograft from the iliac crest^38^. They reported increased bone formation in the β-TCP group compared to the autologous-bone-transplant group (22.90 ± 5.62% vs. 20.87 ± 5.40%), but there was no statistically significant difference between the two groups in this regard^38^. In a subsequent investigation, Janssen et al. reported that the modification of β-TCP granules by embedding them in carboxymethyl cellulose glycerol putty led to the formation of an alternative grafting material that was nearly comparable to autologous bone in handling^39^. Furthermore, no significant differences in bone formation were found via μCT in the histological sections and in the reconstructed bone volume.

Another alternative is allogeneic bone graft, which is already being used clinically for cleft repair. Allogeneic bone grafts customized using CAD/CAM (computer-aided design/computer-aided manufacturing) techniques have been introduced in adult cleft treatment^40,41^. Complete osseous integration and fusion of the grafts in the recipient region were reported. However, the corresponding histomorphometric analysis of the healing process of the human bone allograft in animal research is limited because the human-derived bone substitute must be assessed as xenogeneic transplant in animals, and here the collagen structures still contained can cause immunological reactions^31^.

A sufficient evaluation of calcified tissue and a quantitative analysis of bone formation can be done in human and animal research via μCT to evaluate bone graft healing^34,42–45^. Due to the isotropic voxel sizes and standardized voxel units, the use of volumetric μCT data is appropriate for quantitative analysis, such as the analysis of the bone structural morphology using high-resolution 3D imaging at in vivo and ex vivo laboratory settings^29,46–48^. Bone morphology and calcifications offer a strong native contrast, which allows bone microarchitecture analysis and hard-tissue quantification^49,50^.

Ionizing radiation dose of the μCT could influence the study outcome with regard to animal survival^51^. In comparison to clinical CT scans, the μCT scanning doses in animal research are often relatively higher due to the smaller volumes and lower signal per voxel. In the performed investigation, each rat was scanned eight times in a period of 3 months, in contrast to other longitudinal in vivo studies where animals can receive up to eleven scans over two days without apparent symptoms^52^. Moreover, the present μCT scans were mainly restricted only to the head and skull area which is relatively insensitive to irradiation. During the whole study period each animal received a cumulative radiation dose of approximately four Grey. In this context, Zhai et al. were able to demonstrated that a bony impairment in rats, caused by a single dose of two Grey irradiation, is reversible and will presumably recover completely^53^. Based on the score sheets course of study, no radiation related effects were noticed.

In the present study, the radiological comparison of the three grafting materials generally showed the highest BMD and BV/TV in the synthetic β-TCP substitute, followed by the autologous and then the xenogeneic/human bones. The differences were statistically significant for the comparisons with the xenogeneic/human bone, which had comparatively lower values. During the investigation, an increase in BMD was found in the autologous-bone and synthetic-bone-substitute groups while a slight decrease was found in the xenogeneic/human-bone group. The corresponding BV/TV values were constant for the autologous-bone group but decreased slightly in the human-xenograft group and increased in the synthetic-bone-substitute group. However, most of the changes in bone structure that were recognized during the investigation were not statistically significant.

Nevertheless, the aforementioned changes probably indicate new bone formation with a consistent or decreasing amount of grafting material. In contrast, in all the groups, the bone surrounding the cleft was nearly comparable. Thus, the bone activity appears to be greater in the graft than in the surrounding bone.

Ru et al. has analyzed the bone quality after alveolar defect repair in rats in the context of orthodontic tooth movement^19,20^. However, their findings cannot be directly compared with those of the present study because the defect models and analysis specifications that were used in the two studies differ considerably. On the one hand, Ru et al. examined the bone that was in close proximity to the moving tooth, and on the other hand, the region of interest was further subdivided. In contrast, in the present study, the focus was on the reconstructed jaw section and the surrounding local bone. As a result, there are partially significant differences between the study results. Ru et al. obtained a 15.57–43.73% BV/TV in the control group, 17.50–66.70% in the bovine xenogeneic group, and 17.58–69.06% in the β-TCP group^19^. In contrast, the current values for the grafting materials and the associated surrounding bone were about 54.09 and 96.50% in the autograft group, respectively; about 35.60 and 91.11% in the xenogeneic/human bone group; and about 73.50 and 95.01% in the synthetic-bone-substitute group.

Furthermore, Ru et al. reported a reduction in BV/TV and the corresponding trabecular structures during the first stage of the healing process in all the three groups, followed by a subsequent increase, where the measured values were the highest in the β-TCP substitute group^20^. In the present investigation, the highest values and steady increases in BV/TV and BMD were also found in the synthetic-β-TCP-substitute group. At the latest time from the start of the tooth movement (µCT 3), the difference in BV/TV between the synthetic-bone-substitute and human-xenograft or autograft groups was statistically significant.

Sun et al. histologically investigated the remodeling process of the grafts from the iliac crest for cleft repair in the corresponding artificial defects in rats and found complete graft integration 8 weeks after the orthodontic stress was applied^18^. Thus, they concluded that orthodontic tooth movement into the alveolar cleft bone graft area promoted remodeling of the embedded bone, inducing bone resorption and subsequent deposition. In contrast, in the present investigation, the grafting material was still recognizable in the histopathological analysis even after 12 weeks. Furthermore, the current histological investigation showed a similar amount of persistent augmented grafting material in all the groups after 12 weeks. However, both the autologous and xenogeneic/human bone could be detected with the support of polarization optics due to their already structural fusion with the native bone. In contrast, the remaining synthetic bone substitute material was distinctly recognizable. The strongest new bone formation occurred within the xenogeneic/human bone (79.45%), and the lowest, within the synthetic bone substitute material (5.41%). Furthermore, the present histological findings confirmed the radiological observation that the β-TCP substitute is significantly more compact than the two other types of grafting material. It seems that both bone-based grafting materials more strongly induced new bone formation compared to the synthetic bone substitute, which also apparently less resorbed and integrated into the cleft defect.

## Conclusion

With the limitations of an animal research study, especially the need for assessing the use of a human bone as a xenogeneic grafting material in animal research before being used for such, the use of the β-TCP/HA bone substitute must be questioned. This grafting material for cleft repair was more detectable than the autologous and xenogeneic/human bones even after 12 weeks of healing while the highest activity was found during the healing and orthodontic-tooth-movement periods. Both the autologous and xenogeneic/human bones showed good integration in the cleft defect and bony fusion with the local surrounding bone. However, autograft must still be considered the gold standard due to its higher radiological bone activity. In this context, the additionally applied orthodontic tooth movement seems to have a secondary role in the remodeling process. Further studies have to prove if tissue engineering by combining the xenogeneic/human graft with growth factors can initiate a paradigm shift in cleft repair.

## Data Availability

All data generated or analyzed during this study are included in this published article.
